# A deflectable video camera system for laparoscopic surgery based on shape memory alloy actuators

**DOI:** 10.1038/s41598-025-95898-8

**Published:** 2025-04-02

**Authors:** Malte Pietsch, Konrad Henkel, René Körbitz, Ronny Hüttner, Kai Uhlig, Sascha Bruk, Matthieu Fischer, Jochen Hampe, Andreas Richter, Franz Brinkmann

**Affiliations:** 1 TUD Dresden University of Technology, Chair of Microsystems, Dresden, SN Germany; 2 IPF Leibniz Institute of Polymer Research Dresden, Dresden, SN Germany; 3 Department of Medicine I, TUD Dresden University of Technology, Dresden University Hospital, Dresden, SN Germany; 4 Else Kröner Fresenius Center for Digital Health, TUD Dresden University of Technology, Dresden, SN Germany

**Keywords:** Endoscopy, Laparoscopic surgery, Medical robotics, Shape memory alloys, Smart materials, Gastroenterology, Actuators, Biomedical engineering

## Abstract

Laparoscopic instruments are usually not deflectable and therefore have significant usability limitations in minimally invasive surgery. Flexible-tip devices have been introduced, but they are still controlled by manual Bowden cables or conventional motors and are heavy and expensive. An interesting alternative are shape memory alloy (SMA) actuators, because they are lightweight, cost-efficient and offer the potential for a universal actuator platform for laparoscopic and other endoscopic instruments. This paper presents a laparoscopic video camera with electronic deflection control via thumb joystick. Its actuator unit consists of four SMA wires, whose special tensioning mechanism ensures both optimal preload and length compensation. The camera is tiltable in two degrees of freedom, enabled by a laser-cut Nitinol joint. Based on three assembled units and an automated measurement system, the functionality of the camera system was characterised. Deflection angles of more than 178° per degree of freedom were achieved. At operating frequencies of up to 0.5 Hz, the units showed suitable deflection behaviour. Each remained operational for more than 1200 actuation cycles or 4.4 h of continuous operation. Furthermore, the influence of continuous temperature stimulation on characteristic device parameters was investigated. This work contributes to demonstrating the feasibility of SMA actuators for the electrical control of flexible-tip laparoscopic instruments. Temperature control in particular is a challenge for future work.

**Supplementary Information:**

The online version contains supplementary material available at 10.1038/s41598-025-95898-8.

## Introduction

Laparoscopic surgery, a minimally invasive approach of abdominal surgery, involves the use of a pneumoperitoneum, in which the abdominal cavity is filled with CO_2_ and accessed through small incisions with specialised instruments and the use of rigid video optics to visualise the surgical area^1,2^. Over the past four decades, laparoscopic surgery has revolutionised the field of surgery, providing distinct benefits for patients, including reduced hospitalization times, minimised blood loss, and improved cosmetic outcomes^3,4^. However, conventional rigid laparoscopic instruments pose several challenges for surgeons and are associated with certain drawbacks. These include reduced depth perception and greater ergonomic stress than open surgery, which may contribute to long-term health issues for the surgeons^5^. Furthermore, rigid laparoscopic devices have a limited number of degrees of freedom and cannot be repositioned easily, making it difficult for surgeons to achieve optimal working and viewing angles in areas obstructed by organs^6,7^. Consequently, laparoscopic surgery requires a significantly longer training than open surgery^8–10^. To address the limitations of laparoscopy, several robotic surgical systems were developed. These systems offer high-resolution visualization, improved dexterity, multi-jointed instruments, and ergonomic comfort^8,11,12^. However, they also have several significant downsides, including the loss of haptic feedback for the surgeon as well as high initial (approx. $2.7 million) and maintenance costs (approx. $200,000 per year) required to purchase and operate these systems^13–17^. In addition, each procedure performed with a robotic system requires between $3000 (cholecystectomy) and $17,000 (rectal resection) in disposable and consumable materials^18^. Consequently, although a robotic surgical system offers the advantages of flexible instruments, its cost and size limit its feasibility to only a few specialised centres. To overcome these challenges, there is a need for lightweight and flexible robotic instruments for laparoscopy^19^.

Current laparoscopes with flexible tip mostly use manually driven wires to deflect the tip (e.g., *Olympus Endoeye Flex*®). Robotic-assisted surgery (e.g., *Intuitive Surgical da Vinci*) uses instruments with tension wires driven by electric motors.

This work aims to develop a laparoscopic instrument as a first use case of a universal actuator platform based on shape memory alloy (SMA) actuators made of nickel titanium (Ni–Ti, Nitinol) for a wide range of instruments. The properties and functionality of SMA actuators have already been described and discussed in detail^20–22^. The shape memory effect is based on a temperature-dependent phase transition of certain alloys such as Ni–Ti or Ni–Ti–Cu. These have a low-temperature phase, the martensite, and a high-temperature phase, the austenite. An appropriate temperature change causes a diffusionless, reversible martensitic transformation between the martensite and the austenite. On cooling, the austenite transforms to twinned martensite, which is detwinned when subjected to a certain mechanical stress, resulting in significant elongation of several percent in a wire. Upon heating and corresponding transformation to austenite, the wire contracts and performs mechanical work.

In previous publications, SMA materials are activated via Joule heating^23–25^. This activation does not require an external heat source and is easy to realise electrically. Another advantage of SMA are their intrinsic sensing properties. SMA-actuated micro-grippers with integrated self-sensing capabilities have been successfully developed in previous studies^26^. The integration of sensory feedback into robotic surgical systems has also been shown to enhance surgeon performance^27^. This highlights the potential benefits of SMA-based actuators in surgical applications.

The shape memory effect has the highest energy density of all solid-state actuator effects^28^. SMA actuators are particularly suitable for the use in confined spaces. They are frequently used in the field of soft robotics^24,29^. As wires, they combine flexibility and the very high energy density. In the medical field, some SMA-based concepts exist to actuate laparoscopic instruments. SMA wires are used, e.g., to drive forceps with the antagonistic actuator principle or are combined with dc motors to actuate laparoscopic needle driver jaws^30,31^. Due to their geometry, SMA wire actuators fit well into the installation space of endoscopes and also enable the potential of low-cost roll-to-roll production via multi-material coextrusion.

In this paper, a laparoscopic instrument with a deflectable tip actuated by SMA wires is presented. The developed device features a video camera at the tip and is controlled via thumb joystick. It is driven by a specially developed programmable controller system which enables the acquisition of measurement data from the device. In addition, the first steps have been taken towards implementing cyber-medical assistance features, i.e., the computer-aided control of the joint position and different operating modes. The device was functionally characterised in a specially developed automated test system. Due to the simple mechanical design and the small number of parts, the device concept is also potentially suitable for the production of disposable medical instruments.

## Methods and procedures

### Requirements and constraints

The technical requirements and design for a laparoscopic instrument with deflectable tip and shape memory alloy actuators are based on state-of-the-art laparoscopic instruments and clinical settings. With respect to the current state of the art and the use of SMA actuator wires, there are a number of challenges to consider. Table 1 provides a list of key design requirements. In addition to mechanical design and durability requirements, demands for operating temperatures were also defined based on research and on real-world procedures. The target angle, outer diameter, and working length were derived from standard laparoscopes (*KARL STORZ HOPKINS*® *Telescopes*, *KARL STORZ ENDOCAMELEON*®)^32^. Two bending sections were measured to determine the required bending radius *r*_joint_ (*Ambu*® *aScope Gastro*: 21 mm at 210°; *Olympus*® *PCF-140L*: 25.1 mm at 210°^33,34^. A central task was the assessment of the occurring device temperatures and the protection of the potential patient from electrical voltages. The device temperature may not exceed 50 °C. There is a risk of tissue damage above this temperature^35^. A typical laparoscopic resection in a confined space is the low anterior resection for rectal cancer and takes between 3 and 4 h^36^. A minimum operating time of 4 h is therefore required. An external thumb joystick was chosen as an ergonomic control approach for the electrically operated laparoscopic instrument. To reduce complexity, this initial design does not include a standard biocompatible joint cover.

**Table 1:** Design requirements for the laparoscopic instrument

Parameter	Value
Target angle	≥ |± 90°|
Bending radius	≤ 25 mm
Working length	310–420 mm
Outer diameter	≤ 10 mm
Operation time	≥ 4 h
Device temperature	≤ 50 °C

### System structure

**Fig. 1:**
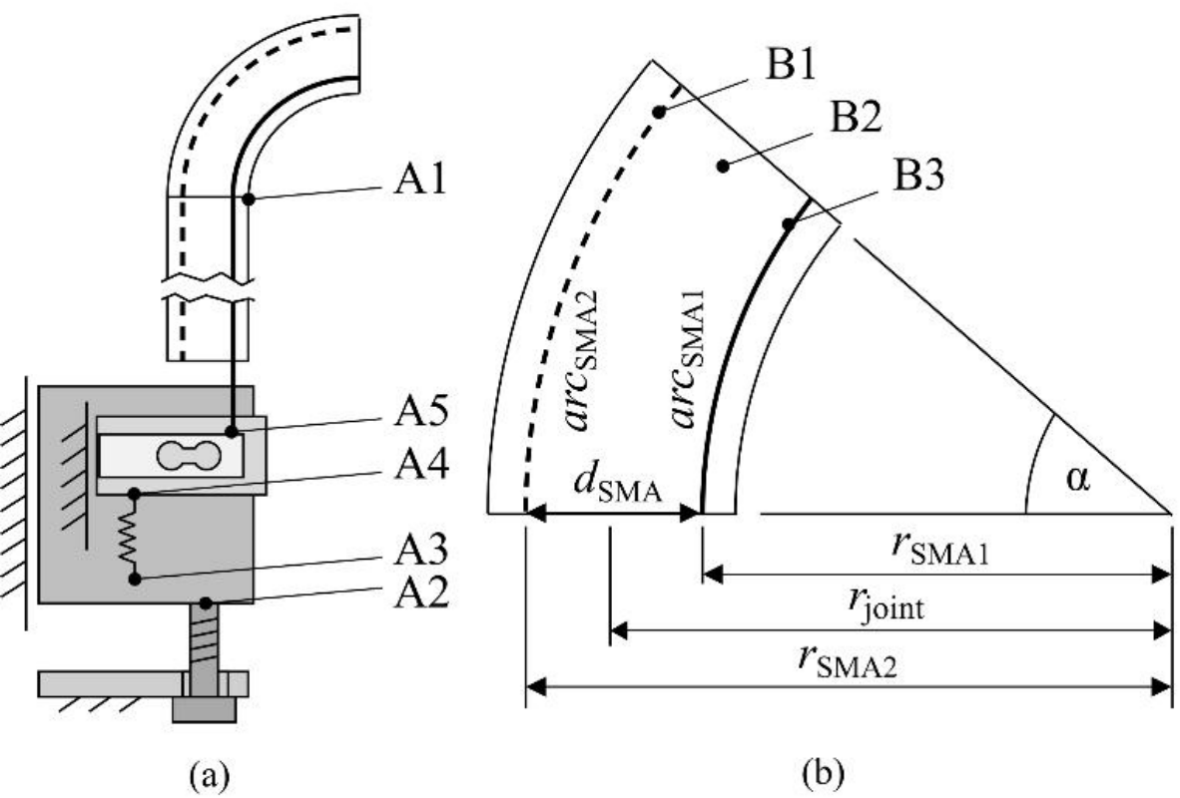
(**a**) Schematic representation of the developed wire tensioning and bending mechanism: (A1) Instrument shaft and flexible tip deflected by SMA wire pair; (A2) Linear guide and setscrew; (A3) Tension spring; (A4) Guide for wire movement; (A5) Force sensor connected to wire. (**b**) Calculation of the required wire length using the angle-dependent length difference: (B1) Inactive wire/antagonist; (B2) Flexible joint; (B3) Active wire/agonist.

The mechanical properties of Ni–Ti based SMA actuator wires change with load and the number of activation cycles. In^37^, it is shown that within 5000 activation cycles at a constant stress of *σ* = 200 MPa, the wire length in the martensitic state increases by 1.4%. To achieve a repeatable shape memory effect, Ni–Ti actuators require a bias stress to detwin the martensite structure after the austenite—martensite transition during cooling. Mechanical tensioning elements are needed to compensate for any wire slack caused by fatigue, transformation induced plasticity (TRIP), tolerances in the assembly process and external loads. TRIP is the irreversible deformation accompanying transformation caused by cyclic thermomechanical stress^38,39^. In addition, the tensioning elements ensure a bias stress at all times. The mechanical tensioners were designed for individual prestressing of the SMA wires and length compensation using tension springs. In addition, a force sensor was used in each case to measure the tensile forces of the respective SMA wires and determine their performance parameters. Fig. 1a shows the developed mechanism in a simplified manner for the unilateral deflection of a flexible laparoscope tip. Four symmetrically arranged SMA wires are routed through an instrument shaft and a flexible joint (Fig. 1a, A1). These wires operate in antagonistic pairs for back-and-forth motion in two degrees of freedom. A linear guide with a setscrew (Fig. 1a, A2) is used to compensate for tolerances during assembly and to adjust the necessary bias stress. Another linear guide (Fig. 1a, A4) is mounted on it, to which the force sensor (Fig. 1a, A5) is attached. The SMA actuator is connected to the force sensor. The tension spring (Fig. 1a, A3) connects both carriages thus tensioning the attached wire.

### SMA actuator layout

The following assumptions were made for dimensioning the required SMA wire length *l*_0_. The actuator life decreases with increasing strain and stress loads^20,37^. An operating strain of *ε* < 0.03 was chosen to realise a robust design. In the following sections, it is assumed that the instrument is in an upright position. The arc length of a wire *arc*_SMA_ depends on the bending radius *r*_SMA_ and the joint angle *α*, as seen in Fig. 1b. The distance between the wires *d*_SMA_ is calculated with the bending radii *r*_SMA1_ and *r*_SMA2_.

1 $${arc}_{\text{SMA}}=\frac{{r}_{\text{SMA}}\cdot \pi \cdot \alpha }{180^\circ }$$

2 $${d}_{\text{SMA}}={r}_{\text{SMA2}}- {r}_{\text{SMA1}}$$

The angle-dependent length difference ∆*l* between inner and outer wire can be calculated using

3 $$\Delta l= \frac{\pi \cdot \alpha }{180^\circ } \left({r}_{\text{SMA2}}- {r}_{\text{SMA1}}\right)= \frac{\pi \cdot \alpha }{180^\circ }\cdot {d}_{\text{SMA}}.$$

The actuator wires within the joint and the shaft maintain a spacing of 7.4 mm. With a target deflection angle of α = ± 90°, the difference in wire length is ∆*l *= 11.6 mm. Therefore, the activated SMA wire must shorten at least ∆*l*_SMA_ = 5.8 mm. The lengthening of the non-active antagonistic wire is provided by the elastic deformation of the wire (minor extend) and the bias spring (major extend). The required wire length is calculated according to

4 $${l}_{0}= \frac{{\Delta l}_{\text{SMA}}}{\varepsilon }.$$

With *ε *= 0.01 to 0.03, a nominal length of the SMA wire *l*_0_ = 193 to 580 mm is required. In the developed system, a wire length of *l*_0_ = 440 mm was selected. The working strain was calculated to *ε *= 0.013 by applying (4). The effective working length of the instrument of 347 mm is within the specified design requirements.

SMA wires with an austenite start temperature of 70 °C were selected for the actuator assembly (*Flexinol*®, *Dynalloy Inc.*). To ensure a complete detwinning in the martensite state, a minimal bias stress of *σ*_min_ = 70 MPa is required^40^. For the selected wire with a diameter of 100 µm, this corresponds to a tensile force of *F*_min_ = 0.57 N. Due to the previously mentioned factors that can cause wire slack, the initial bias force was set to *F*_bias_ = 0.8 N using the tensioning mechanism presented in Fig. 1a. The springs (Fig. 1a, A3) have a spring rate of *k* = 0.149 N/mm and an unloaded length of *l*_spring_ = 16.6 mm^41^.

### Flexible joint and shaft section

Requirements for a deflectable tip for laparoscopic devices include degrees of freedom, outer and inner diameters, as well as robustness, and low deflection forces. In addition, electrical and thermal insulation must be realised to protect potential patients. Based on these criteria and those listed in Table 1, an appropriate laparoscopic joint was developed. The design and manufacturing process based on a standard semi-finished Nitinol tube and laser-cut flexure hinges are described in detail in^42^. With the intended maximum deflection of 90°, the length of the joint could be reduced from 70 to 44.6 mm. Without the bases, resulting in an active joint length of 39 mm, a smaller bending radius *r*_joint_ = 22.3 mm is achieved. This value is similar to the value of a current bending section *Olympus*® *PCF-140L* (25.1 mm). Based on *r*_joint_ and *d*_SMA_ the wire bending radii are *r*_SMA1_ = 18.6 mm and *r*_SMA2_ = 26 mm.

A segmented approach was chosen to electrically isolate and guide the SMA wires within the flexible joint section. The design requirements for the development of the segment geometry were to minimise the influence on the Ni–Ti joint and the additional deflection force, and to prevent friction and jamming between the inner and outer parts of the joint.

**Fig. 2:**
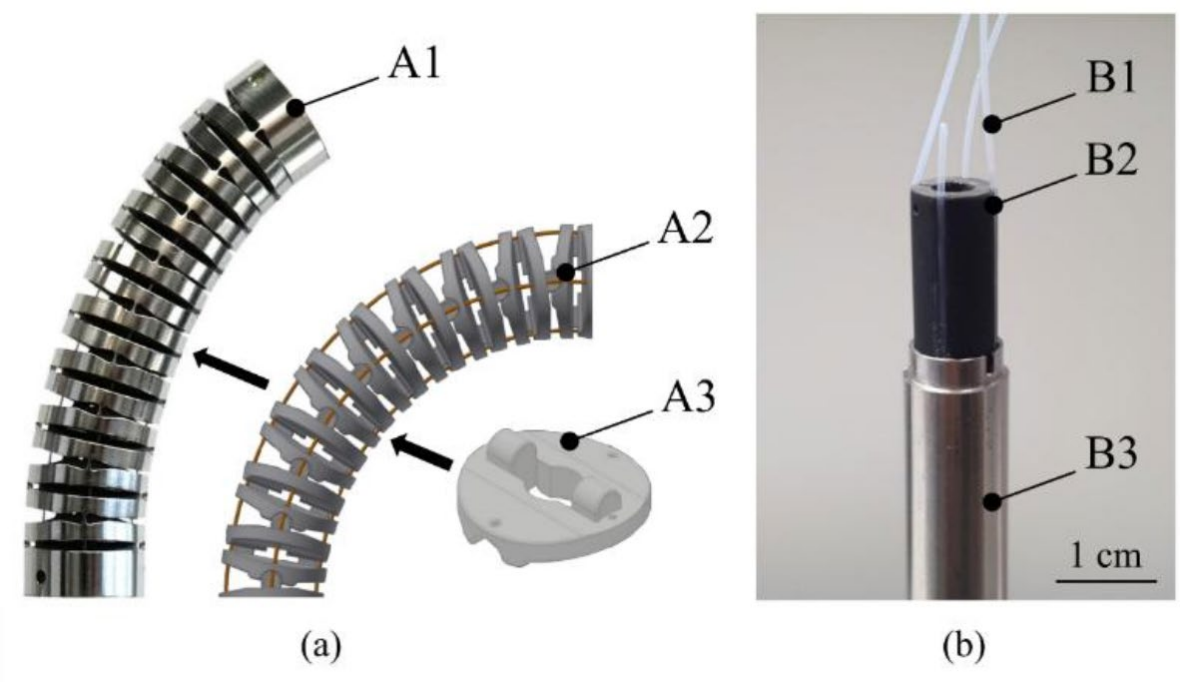
(**a**) Instrument joint assembly: (A1) Finished assembly of the bending section with flexible Ni–Ti joint; (A2) CAD model of the inner stacked segments for insulation and guiding of the wires; (A3) CAD model of a single segment with guide holes and rolling surfaces. (**b**) Guiding and sheathing in the instrument shaft: (B1) PTFE tube; (B2) 3D printed inlay; (B3) Stainless-steel tube.

To achieve this, an inner segmented guiding section consisting of 19 identical injection-moulded polyoxymethylene components was designed (Fig. 2a, A2). The components are stacked and alternately rotated by 90° to allow for circumferential movement. Several versions of the stacked segments were designed, with the articulation points ideally aligned with those of the outer Ni–Ti bending section. The joint geometry has discrete articulation points so that it follows the bending line of the outer joint section and keeps a consistent bending length with the outer joint. The segment shape and size provide a 300 µm clearance to the outer Ni–Ti component to additionally prevent jamming. An air gap is also provided between the segments at 90° deflection to prevent wire shearing and enable optimal guidance.

Friction at the contact points between wire and segment, and between segment and segment, can potentially induce jerky motion during small and non-continuous wire movements. This unwanted effect occurs with isolated segment joints. However, in the selected joint configuration, it is masked by the spring force of the solid Ni–Ti joint and is therefore less relevant. If jerky motion does occur, potential solutions may include reducing the static friction value of the material pairing with polytetrafluoroethylene (PTFE) coating or lubricants, or by employing control algorithms that take static friction into account. The deflection force for the joint and inner segments was measured separately on all four SMA wires in the absence of an antagonist to be *F*_90°_ = 0.59 N (*σ* = 0.037 N; *n* = 12).

The complete assembly is shown in Fig. 2a. The flexible Ni–Ti joint is attached to a stainless-steel tube. The wires in the tube area are sheathed with PTFE tubing. This prevents electrical short circuits to the steel tube, provides thermal insulation, and minimises friction in the shaft. The PTFE tubes are secured by grooves in an insert element, as shown in Fig. 2b.

### Complete assembly

**Fig. 3:**
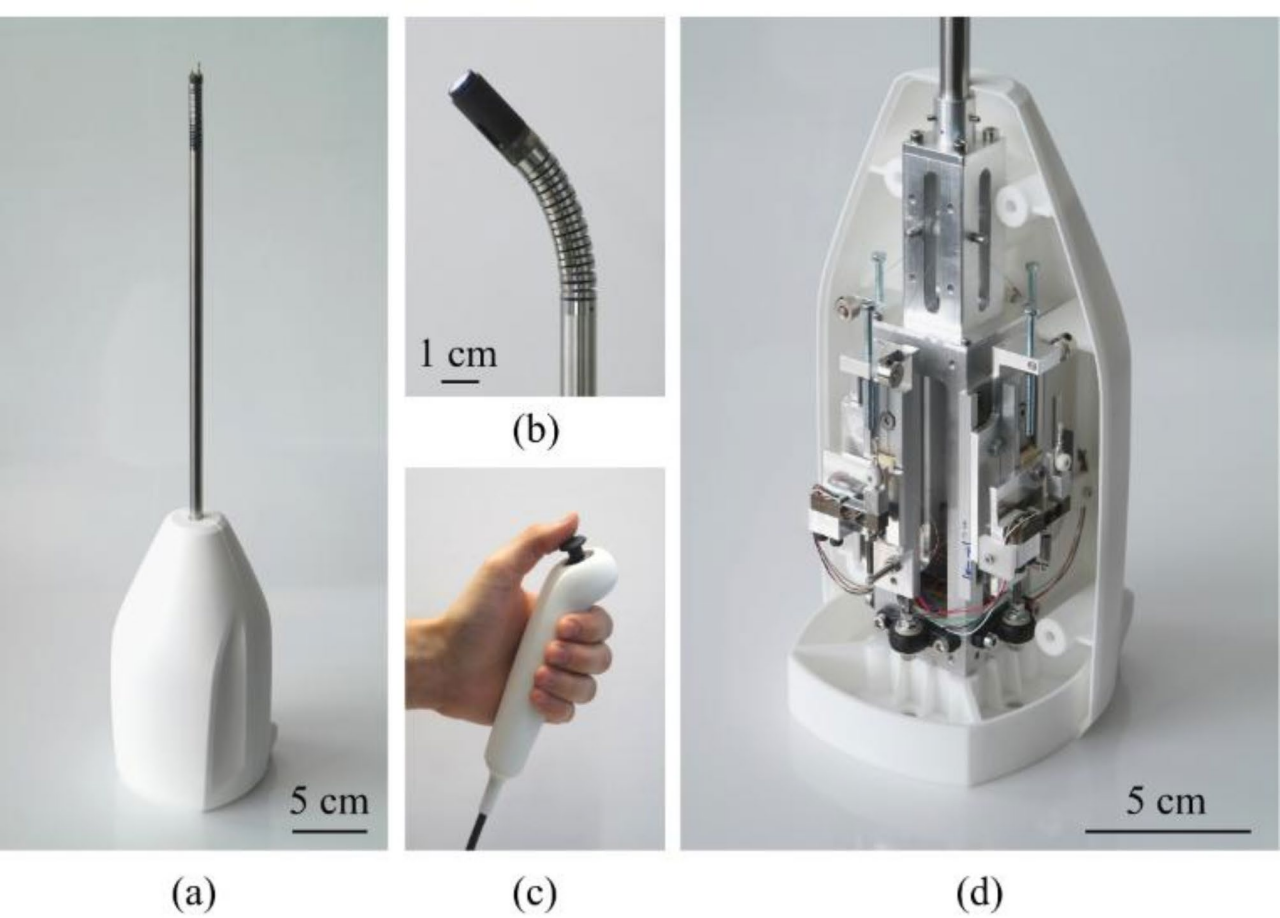
Overview of the developed instrument: (**a**) Complete device unit; (**b**) Flexible joint with video camera; (**c**) Remote control thumb joystick; (**d**) Opened instrument body.

**Fig. 4:**
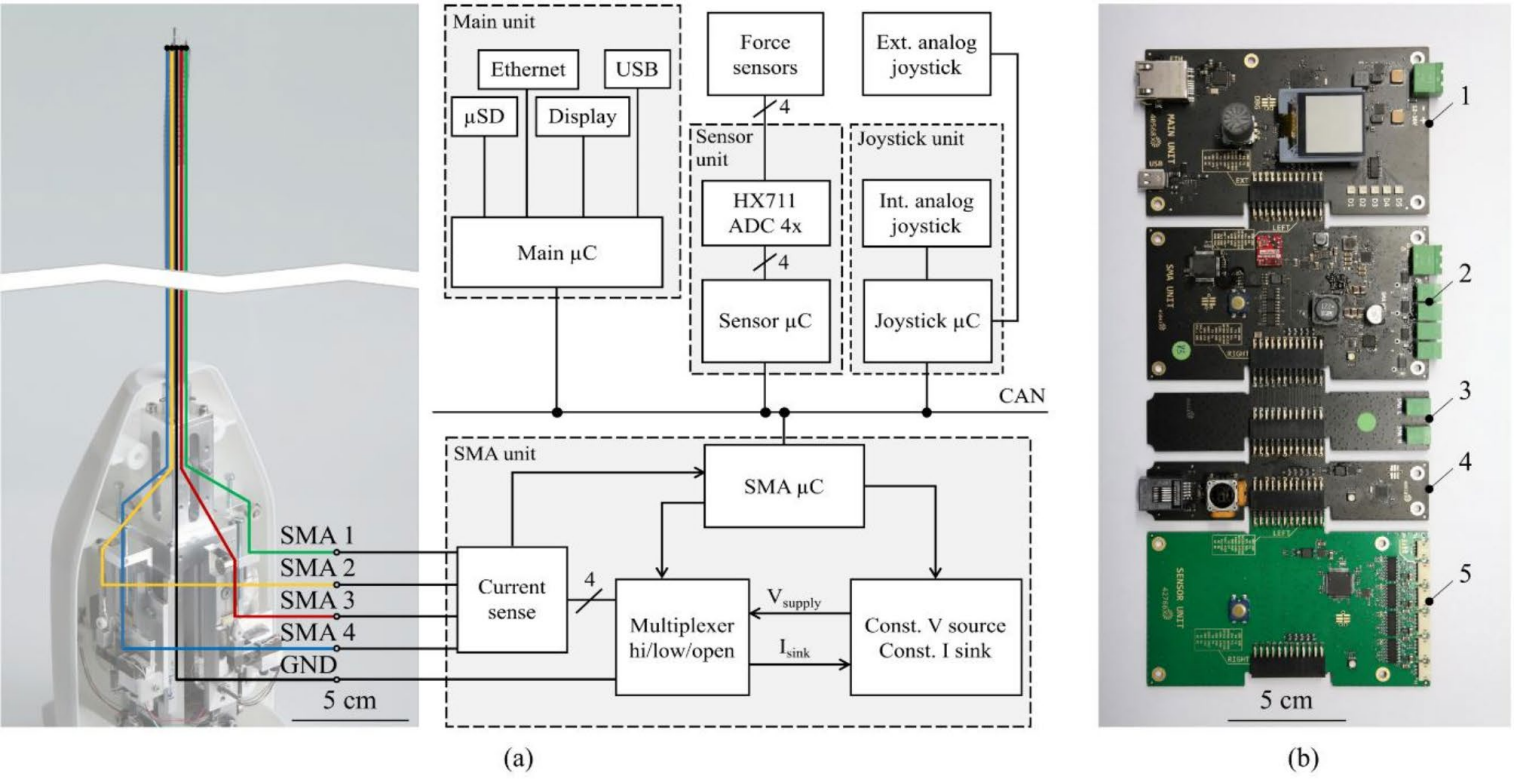
(**a**) Schematic view of the wiring and control electronics. (**b**) Control electronics: (1) Main unit; (2) SMA unit; (3) Additional breakout board; (4) Joystick unit; (5) Sensor unit.

An *OC0SA10* image sensor in combination with the *OH0130* ASIC-based board from *Omnivision* is used for the camera in the deflectable instrument tip^43,44^. The video camera provides 800 × 800 pixels, a depth of field ranging from 5 to 100 mm and a field of view of 120°. The camera is mounted on the end of the joint via an adapter and the video cable was routed through the inner lumens of the joint segments and the shaft inlay. The complete instrument is shown in Fig. 3. At the distal end there is the flexible joint equipped with the video camera. The SMA wires run through the joint and the connecting tube into the instrument body. Upon exiting the tube, they are deflected twice using pulleys to align them with the tensioners (see Fig. 4a). The body contains the main assembly with the force sensors, the tensioning mechanisms and electrical connections. Mechanical fastening and electrical contacting of the SMA wires are provided by crimp sleeves and cable lugs. A device housing was designed and 3D printed by *WOLFRAM Designer und Ingenieure* (Dresden, Germany). It consists of a base, on which the mechanics are mounted, and two housing shells which optionally enclose the mechanics. The electrical connection to the outside is made with three round subminiature connectors.

### Electronic design and control

**Fig. 5:**
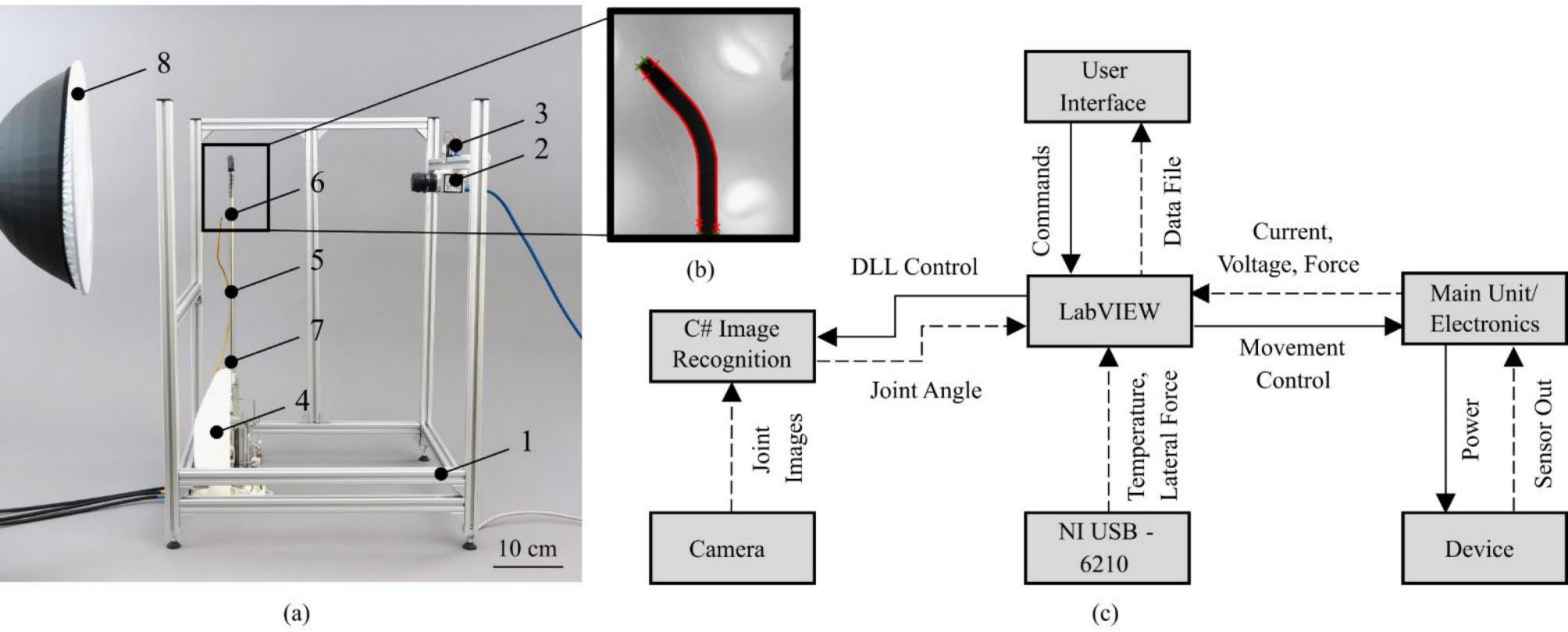
(**a**) Experimental setup: (1) Aluminium frame; (2) Industrial camera; (3) Lateral force sensor; (4) Laparoscopic instrument with wire force sensors; (5) Instrument shaft; (6) Upper temperature sensor; (7) Lower temperature sensor; (8) Backlight. (**b**) Camera view for angle detection with detected contour (red). (**c**) System structure, information and energy flow of the experimental setup.

For the control of the SMA-driven instrument, a reusable modular electronic system shown in Fig. 4b was developed by *Contronix GmbH* (Radebeul, Germany). The electronics consists of four active modules, which communicate via a CAN bus: main unit (Fig. 4b, 1), SMA unit (Fig. 4b, 2), joystick unit (Fig. 4b, 4), and sensor unit (Fig. 4b, 5). The main unit is the central computing unit (see also Fig. 5c). It collects the data of the other modules and handles the communication with the external PC. It receives control commands and operation modes via USB as well as the readings of the force sensors and the electrical feedback of the actuators from the other modules. The microcontroller uses the data to calculate the target currents for the four individual SMA actuators. The SMA wires have a common ground and are powered alternately via a multiplexer on the SMA unit (Fig. 4b, 2). Simultaneous control of different wires with different currents is also possible. A copper wire provides the ground connection and is routed through a central lumen to the tip of the device where all four SMA wires are connected (see Fig. 4a).

By applying voltages to the cable lugs in the instrument body, the respective currents flow through the SMA wires to the central grounding point in the tip. A configuration without a central ground wire is also possible, in which case the return currents are split between the other SMA wires. However, this leads to parasitic heating and requires higher voltages, as the specific electrical resistance of Ni–Ti is much higher than that of copper. The actuator system can be controlled via line commands or joystick input. In stand-alone mode, the control currents are calculated based on the position of the joystick. In this work, an open-loop current control was used, but in perspective, a force feedback closed-loop control can also be applied. The force sensor (*TesT GmbH: 342.20N*) outputs are processed by the analog-to-digital converter (*Avia Semiconductor: HX711*) of the sensor unit at 24 bits and 72 samples/s^45,46^. The raw values are converted to millinewtons by the microcontroller.

The hold-function, a first digital assistance system, was implemented on the main unit to demonstrate the advantages of the smart actuator approach over conventional laparoscopic drive systems. By pressing the joystick, the electrical current values are stored and maintained to hold the active joint position even if the user releases the joystick.

### Experimental setup

To validate the smart actuator concept, a dedicated experimental setup was developed (see Fig. 5a). It consists of a frame made of aluminium profiles (Fig. 5a, 1) on which an industrial camera (Fig. 5a, 1) (*Basler AG*: *a2A2590-60ucBAS*, Lens: *C125-0618-5M-P*), a force sensor (Fig. 5a, 3) (*TesT GmbH*: *342.5N*) and the instrument (Fig. 5a, 4) are mounted^45,47^. Two PT1000 temperature sensors were attached to the instrument’s stainless-steel tube, 1.5 cm from each end, to measure the operating temperature at the surface (see Fig. 5a, 5–7). To improve the contrast of the camera image, a backlight (Fig. 5a, 8) was used. The industrial camera is placed in a well-defined position relative to the instrument joint and captures its deflection in a fixed plane at 60 frames/s at a resolution of five megapixels. The force sensors in this setup and in the tensioning mechanisms of the device were calibrated according to the manufacturer’s specifications.

A schematic representation of the system structure of the experimental setup is shown in Fig. 5c. The setup is controlled by a *LabVIEW* program, without using the external joystick. It governs the experimental sequence, collects data from all electronic subsystems and sends control commands to the main unit via a PC interface. In addition to the control electronics, the subsystems include a *NI DAQ USB-6210* for measuring surface temperatures and lateral joint force and the camera interface for image-based measurement of the joint deflection angle^48^. The video stream of the industrial camera is analysed in real-time by an algorithm that detects the contour of the laparoscope in each image and calculates the current joint deflection (see Fig. 5b). The detected contour is approximated as a polygon using functions from the *OpenCV* library^49^. The principle of the angle calculation is a partial integration of the interior angles of adjacent edges of the polygon.

### Experimental sequence

To evaluate the performance parameters of the developed system using the SMA actuator platform, experiments were performed after assembly and fine tuning of the bias tension. The experiments aimed at key operating properties including system dynamics, the characteristic deflection behaviour of the antagonistic wire system and overall fatigue behaviour. In addition, the curves of the operating temperature and different system characteristics (driving forces, currents and electrical resistance of each SMA wire) were examined.

**Fig. 6:**
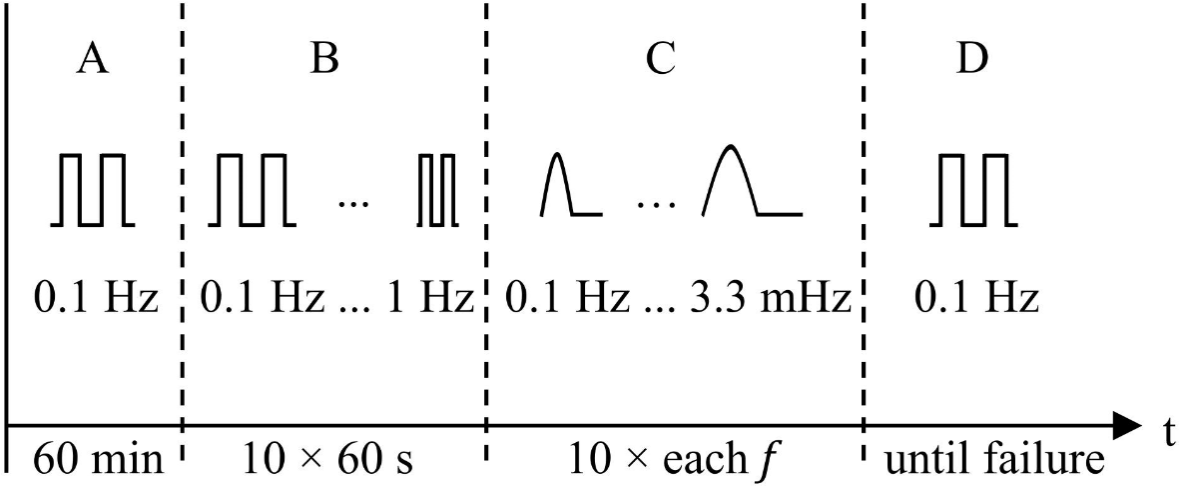
Experimental sequence and schematic duty cycle of one active SMA wire for each device test run: (**A**) Warm up; (**B**) Dynamic behaviour; (**C**) Characteristic hysteresis; (**D**) Fatigue test.

To achieve a specified maximum deflection of 90°, the maximum current value per wire was set to *I*_HIGH_ = 350 mA. This corresponds to 175% of the recommended current specified by the manufacturer for a wire diameter of 100 µm and is still within the permissible current range. In the inactive state, the wires were still supplied with a current of *I*_LOW_ = 40 mA so that their resistance could continue to be accurately measured. Measuring the resistance values in all phases of actuation is important as it provides sensory feedback for future applications such as control and deflection detection. Only one pair of the available wires was used in each of the experiments, so that only one degree of freedom had to be measured by the optical measuring system. A complete activation cycle includes the activation of one wire, the simultaneous deactivation of its antagonist, and the subsequent switch between the two sides, i.e., the symmetrical movement of the joint to one side and back to the other. In general, there are two relevant measurement results for the tests, one for the positive (+) and one for the negative (−) deflection direction of the joint. The entire measurement sequence, illustrated in Fig. 6, consists of four main sections, each targeting distinct characteristics of the SMA-driven instrument. The figure illustrates the qualitative current of a single wire. The wire is activated for half of an activation cycle. The other half of the time, the opposite wire is activated, meaning that the total current input of the device is constant.

In section A, the antagonistic wires were alternately powered for 60 min with a symmetrical square-wave current *i*_A_(t) switching from *I*_HIGH_ to *I*_LOW_ at a frequency of *f* = 0.1 Hz. Using this frequency, a constant final deflection of the joint is expected. This amounts to 360 complete activation cycles per wire. According to Fumagalli et al.^20^, a stable and cycle-independent behaviour can be expected within this number of cycles. The phase transformation and thus the actuator function of the Ni–Ti wires is indirectly controlled by the forced Joule heating effect and passive cooling. This causes the overall device temperature to rise during operation until a thermal equilibrium is reached. The objective of this section was to warm up the device until a static thermal operating point was reached so that static system performance could be measured.

In section B, the device temperature was maintained at the previously determined static thermal working point. Initially, the active wire pair was cyclically powered to both sides with symmetrical square-wave currents identical to section A, *i*_B_(*t*) = *i*_A_(*t*). To investigate the general frequency-dependent deflection *α*(*f*) of the joint in a frequency range from *f* = 0.1 to 1 Hz, the cycle period was then decreased by 1 s after every 60 s until ten frequencies were recorded.

In section C, in contrast to before, the two SMA wires were alternately powered with a sinusoidal half-wave of increasing period duration (*i*_C_(*t*) = *I*_HIGH_ sin(2π*t*/*T*); *T* = 10 s; 30 s; 120 s; 300 s; *n* = 10). This is done in order to record values across the entire available current range and identify their relation to the output deflection. The sinusoidal current has a lower root mean square (RMS) value *i*_C,RMS_ than the alternating square-wave currents *i*_B,RMS_. To minimise the influence of the resulting temperature changes during this section, the actuation was therefore paused after section B until a new constant operating temperature was reached. Each cycle duration was repeated ten times and standard deviations were determined. Afterwards, the maximum lateral force at the joint tip *F*_⊥_ in neutral position (*α* = 0°) was measured. This force is available to surgeons to push tissue aside if necessary. One SMA wire was powered with *I*_HIGH_ until a constant lateral force was measured. This was done five times utilizing the pair of wires not previously used.

In section D, a total number of cycles until failure was determined. The device was again driven with square-wave currents (*i*_D_(*t*) = *i*_A_(*t*)). For the total number of cycles achieved, the evaluation script counts both the rising edges of the input current and the zero crossings of the deflection angle captured by the camera.

The measurement series was performed a total of three times, each time with new wire pairs and bending section. After data acquisition, a *Visual Basic Script* was used to analyse and plot all measured data. Depending on the sampling rate, a moving median of two to twelve points was used to help analyse the results.

## Results

In the following, the measured values of one assembly unit are presented in detail. Subsequently, deviations from the other runs are presented.

### Warm up

The maximum surface temperatures during warm up were determined and the associated effects were analysed. The operating behaviour of the laparoscopic device was observed directly after assembly starting from the *cold state* (ambient temperature *ϑ*_0_ ≈ 22 °C).

The course of the wire forces *F*(*t*), the surface temperatures *ϑ*(*t*), the electrical resistance *R*(*t*) and the deflection angle of the joint *α*(*t*) were measured. In addition, the maximum wire forces *F*_max_(*t*) and the maximum deflection *α*_max_ (*t*) were recorded. For selected cycles, the input step response was evaluated in detail (see Fig. 8).

**Fig. 7:**
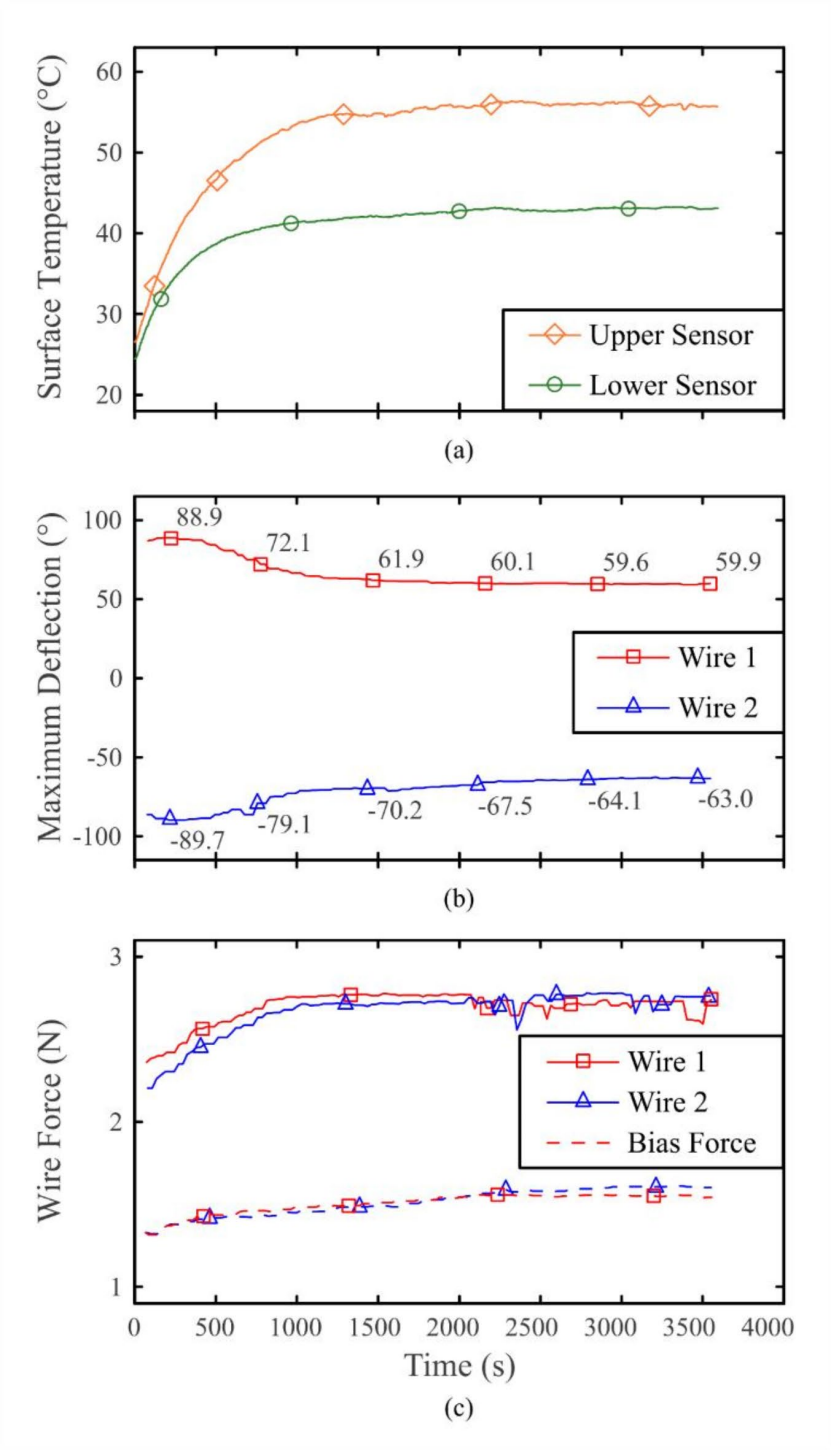
The maximum values of the performance parameters show an asymptotic increase in device temperature and required actuator force, as well as an initial increase in achievable deflection, followed by a decrease in deflection to a stable system behaviour starting at approx. 2300 s (i.e., 230 activation cycles): (**a**) Graph of shaft surface temperature. (**b**) Graph of maximum deflection. (**c**) Graph of tensile forces of the antagonistic wire pair.

The measured temperature follows an asymptotic trend (see Fig. 7a). After approx. 2300 s the temperature at both the tip and the bottom of the instrument shaft was found to be constant. A final temperature of 55.7 °C was measured at the upper sensor and 43.3 °C at the lower sensor.

The maximum achievable deflection rises directly after start up from initial + 83.4° and − 85.6° respectively, to global maxima of + 88.9° and – 89.7° within a short amount of time (200 s; 20 cycles) (see Fig. 7b). Subsequently, an asymptotic decrease of the deflection to constant values of + 60° and – 63° can be observed. This behaviour applies to both sides of the joint, with one side achieving higher values than the other. The bias tension of the relaxed wires, as shown in Fig. 7c, increases and the maximum wire forces rise to constant values of *F*_1,2_ = 2.8 N.

**Fig. 8:**
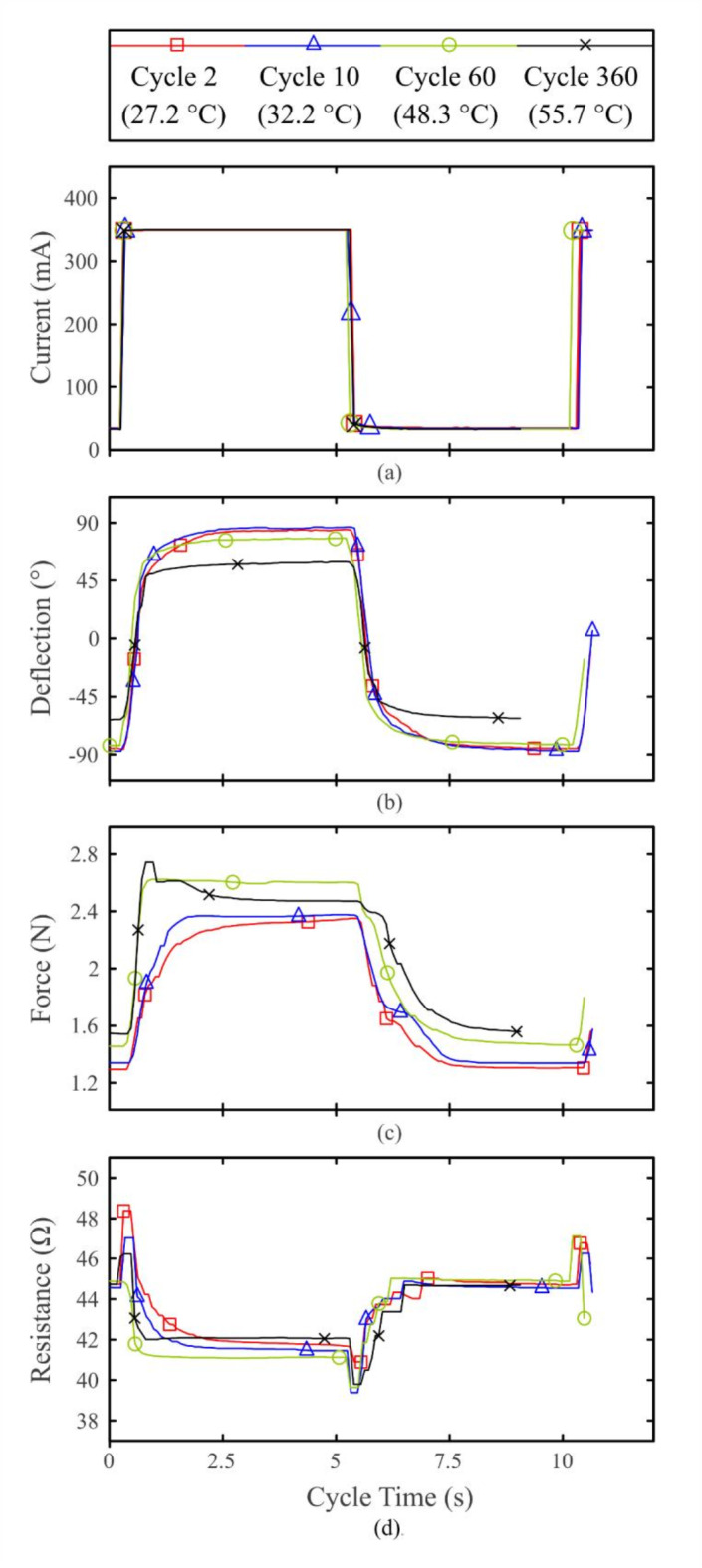
The graphs of selected activation cycles of a single wire show a characteristic behaviour from which the state of the system can be determined. The respective temperatures at the upper sensor are stated for each cycle. (**a**) Excitation current. (**b**) Overall joint deflection. (**c**) Tensile wire force. (**d**) Electrical resistance.

Fig. 8 shows in detail the driving current, deflection, force and resistance graphs of a single wire at specific cycles. From these results, the response times and the deflection latency of the system can be determined. Furthermore, the already observed variation of the maximum values over time as well as cycle-dependent changes of the waveforms are reflected here.

In response to a stable square-wave current (see Fig. 8a), the time delay of the deflection is shown in Fig. 8b. The deflection process is characterised by a short upward slope followed by a plateau phase. Fig. 8d shows the electrical resistance readings, with two different plateau values recognizable, an activated austenitic plateau at approx. 42 Ω and a deactivated martensitic plateau at approx. 45 Ω.

### Dynamic behaviour

**Fig. 9:**
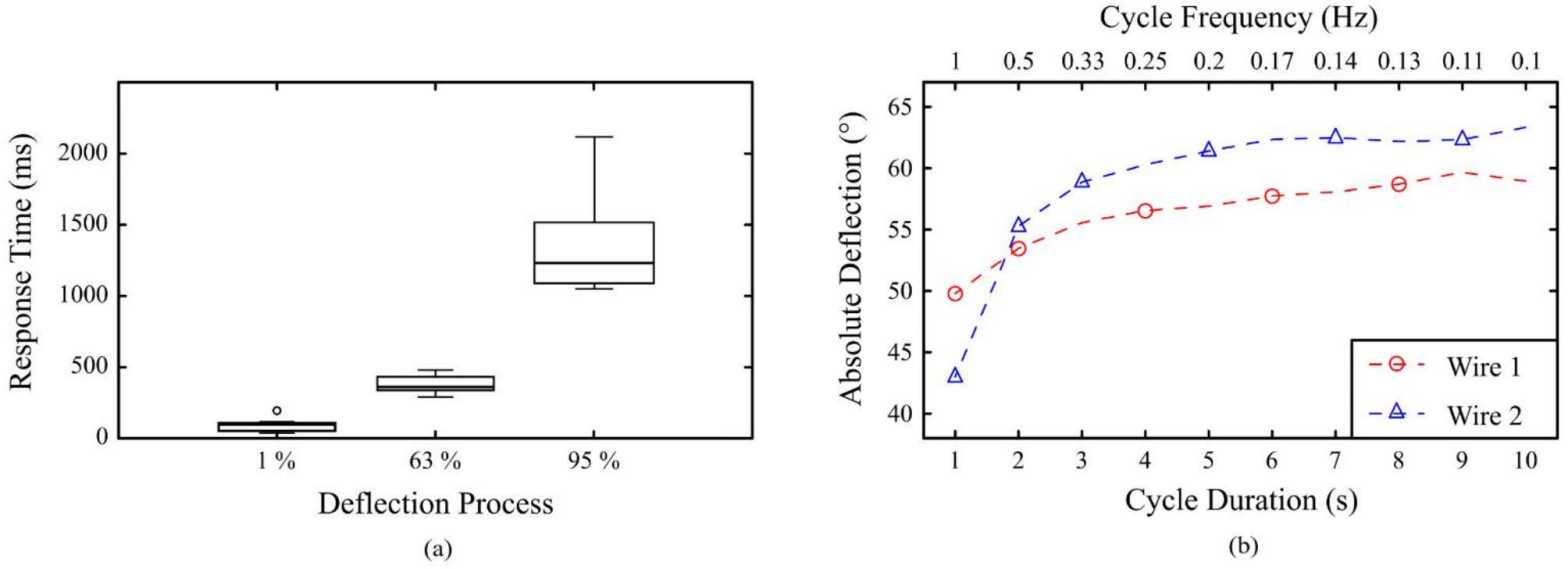
(**a**) The response times of all assemblies to an electrical input show fast reaction, with the deflection process slowing down towards the maximum possible angles. (**b**) The graph of the maximum possible deflection *α*(*f*) shows a nonlinear correlation with the duration of excitation. Below a cycle frequency of 0.5 Hz, the maximum values are reliably achieved.

Following the initial experimental section, the subsequent focus was on the dynamic behaviour of the SMA driven instrument. The time required by the joint to respond to a stimulus is derived from the results of the previous section. The cycles 2, 10, 60 and 360 of each assembly were analysed. Fig. 9a shows the collected response times. The median values of the respond times of all assemblies were *τ*_1_ = 77 ms until the start of deflection, *τ*_63_ = 334 ms until 63% of the maximum deflection was reached, and *τ*_95_ = 1217 ms until 95% of the maximum deflection was reached.

The results in Fig. 9b show an asymmetric response. At cycle frequencies below *f* = 0.11 Hz, the maximum deflections of + 59.7° and − 63.3° were achieved. Up to a frequency of *f* = 0.17 Hz, these values stay consistent with very small errors. A decrease in deflection becomes evident with further increase in frequency.

For both directions, the measured cut-off frequency, at which 90% of the maximum deflection is no longer reached in time, was *f*_c_ = 0.5 Hz. With high deflection frequencies of 1 Hz, 83–68% of the initial deflection performance was still achieved. From the measured values, a maximum actuation speed of approx. 185°/s can be calculated.

### Characteristic hysteresis

**Fig. 10:**
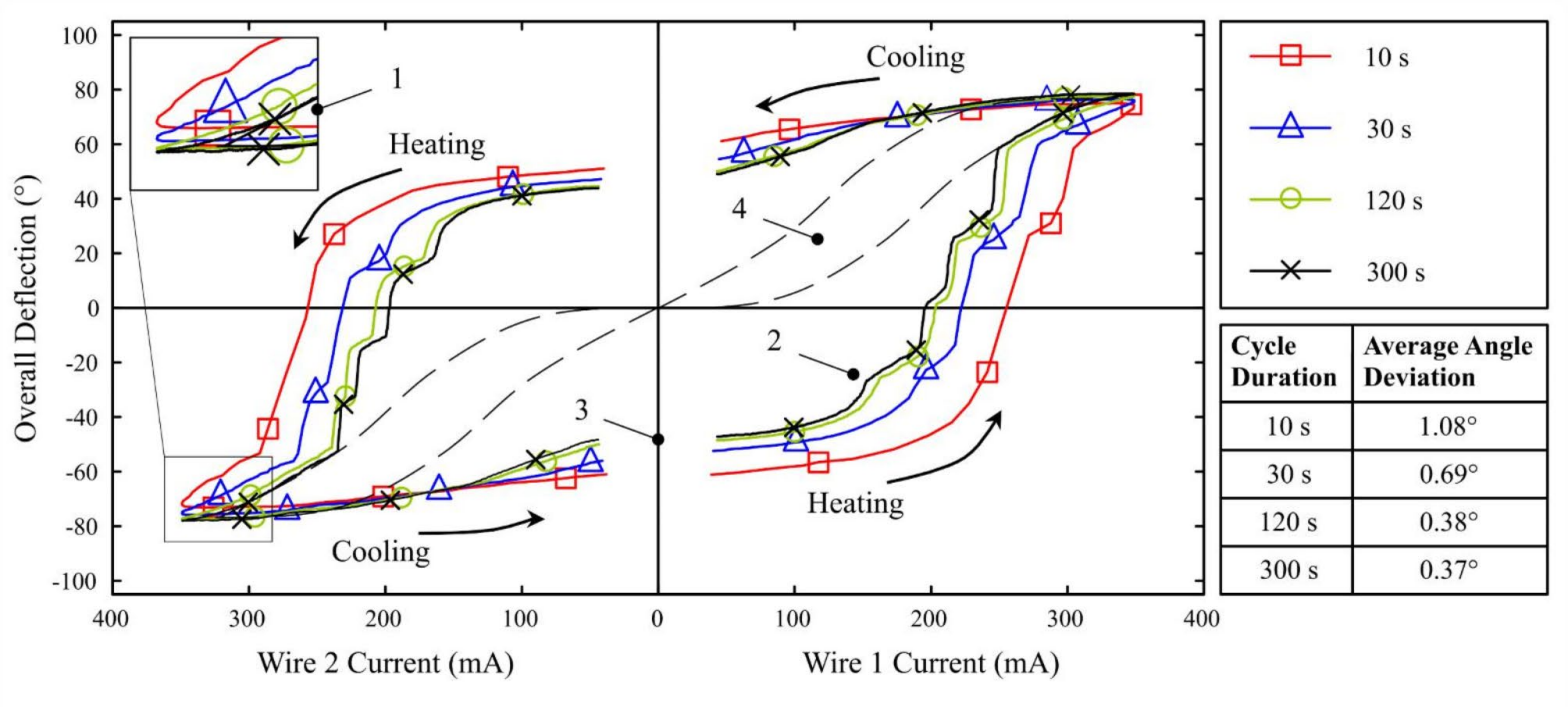
The averaged responses of joint deflection to different cycle durations of the input current for each actuator wire shows expected characteristics: (1) Frequency-dependent maximum deflection; (2) Jumps in deflection; (3) Residual deflection or hysteretic errors at deactivated currents; (4) Typical symmetrical-antagonistic SMA shape. Ten iterations of each period duration also provide an indicator for positioning accuracy.

The averaged results of all measurement points of each cycle duration at a constant upper temperature of *ϑ*_C_ ≈ 45 °C are shown in Fig. 10. The graph shows the bi-directional deflection of the instrument joint against the activation currents of both wires. Due to the symmetry of the mechanical design and the method of voltage application, a diagram mirrored at zero current was chosen. Wire 1 deflects the joint in the positive direction as the current increases, whereas an increasing current in wire 2 results in a negative deflection. By applying alternating sinusoidal half-wave currents of increasing cycle durations, the distinct hysteretic behaviour becomes apparent and dynamic and quasi-static properties can be determined. As before, during the active control of one wire, the other wire was maintained at a constant current of *I*_LOW_ = 40 mA, making no readings appear below this value. The curves have a loop shape reaching their maximum deflection at the highest excitation current. As the SMA wire heats up, there are abrupt changes in the deflection around the zero position (Fig. 10, 2). In the experiments this was observed as a snapping motion of the joint. Two typical hysteresis curves of SMA wires (Fig. 10, 4) are shown in the diagram as dashed lines and serve as reference points. Owing to the electromechanical interconnection, they manifest as a point-symmetrical twin curve. The SMA-driven instrument displays a low average position deviation at 300 s cycle duration. This implies that, for any given current, the resulting deflection has an average deviation of only *σ* = 0.37°.

At the end of this section, the lateral force of the instrument tip was measured. This resulted in a maximum lateral force of *F*_⊥_(350 mA) = 0.167 N (*σ* = 0.002 N; *n* = 5).

### Fatigue test

Two failure patterns were observed during fatigue testing of all three units: First, the fracture of flexural hinges near both ends of the Ni–Ti joint, resulting in a minor degradation of the overall function (see Fig. 10, 2). Second, the breaking of an actuator wire, resulting in system failure. This occurred after 1713, 1428 and 1242 deflection cycles, corresponding to continuous lifetimes of 5.7 h, 4.9 h and 4.4 h, respectively. No failures were observed at the electrical connection points, not even at the mechanically stressed crimp sleeves. The symmetry of the deflection, i.e., the preferred direction of the joint, varied for each assembly. Table 2 summarises the collected results of all three assemblies.

**Table 2:** Design requirements and achieved operating properties at maximum currents of 0.35 A

Parameter	Target	Assembly 1	Assembly 2	Assembly 3
*α*_max_ (Maximum joint deflection angle)	≥ |± 90°|	+ 88.9°, − 89.7°	+ 93.3°, − 93.9°	+90.9°, − 91.8°
*τ*_1, 63,95_ (response time to input)	n/a	(30/244/1180) ms	(59/398/1217) ms	(93/317/1328) ms
*f*_c_ (cut-off frequency)	n/a	0.5 Hz	0.25 Hz	0.5 Hz
*t*_max_ (Operation lifetime)	≥ 4 h	5.7 h	4.9 h	4.4 h
*ϑ*_max_ (Maximum surface temperature)	≤ 50 °C	56.4 °C	56.3 °C	59.3 °C
*F*_1,2.max_ (Maximum wire forces)	n/a	2.8 N, 2.8 N	2.8 N, 2.7 N	2.7 N, 2.6 N
*F*_⊥_ (Lateral force)	n/a	0.17 N	0.21 N	0.19 N

## Discussion

Compared to other SMA-driven instruments, this actuator setup is characterised by a much more expedient antagonistic arrangement of the actuator wires. The novel combination with tension springs results in a capable actuator platform for laparoscopic instruments that demands to be investigated and successfully applied.

The mechanical design requires few parts, such as commercially available SMA wires and simple tensioning mechanisms. Future device generations will no longer require the moving parts and sensors installed in the instrument body. Significant cost reductions can be anticipated in future iterations through design simplification and mass production. The inherent advantages of SMA actuators, such as their simplicity and compact size, will lead to a more cost-effective solution compared to traditional mechanical systems.

The hysteretic behaviour and the quasi-static properties of the relationship between input current and deflection output of the antagonistic SMA circuit are of particular significance with regard to electronic control for cyber-medicine and assistance functions. Results such as those shown in Fig. 10 demonstrate the good reproducibility of the target angles with repeated application of a specific electric current. Due to the complex mechanical structure surrounding the wires, nonlinear properties such as friction and backlash are introduced to the results in form of distortions, creating their own characteristic features. Most prominent are the two opposite peaks (Fig. 10, 1) and the points of zero crossings. The interpolated intersections with the y-axis (Fig. 10, 3) indicate the residual deflection or hysteretic errors caused by the mechanical backlash, friction losses and ultimately the integrated camera cable, which acts as an additional damper in the bending section. The joint exhibits a snapping movement, characterized by discrete, uniformly distributed steps that correlate with the degrees of joint segmentation, particularly visible at the high measurement point densities and advanced lifetimes of the slower cycles. This behaviour can be attributed to the bistable tipping points of the already worn Ni–Ti joints under load. While the solid Ni–Ti joint shows promising deflection forces, future developments should prioritise its robustness to achieve more activation cycles with a uniform deformation behaviour. Friction losses can be further reduced by increasing the clearance for the camera cable, which has been identified as the main cause of residual deflection. In addition, coating the segmented wire guide in the joint assembly with PTFE can help to further reduce friction hysteresis.

The measurement data allowed to determine the effect of temperature-dependant phase transition properties of the SMA actuators on the operating behaviour and to investigate the changing SMA resistance under temperature stimulation. In accordance with the literature, we observe a stable and cycle-independent behaviour within the first 360 activation cycles^20^. All three sets of experiments showed the same characteristic behaviour. The measured lateral forces are comparable to those of other lever-assisted SMA mechanisms found in the literature^50^. With the exception of section C, the highest possible loads were applied by the square-wave activation currents throughout the whole test sequence. The operating times of the units tested were within the expected range and the total number of cycles achieved is similar to the range of constant-stress tests performed in other work^37^.

Looking at the deflection angles of the laparoscopic instrument, one side achieved higher values than the other. This suggests the presence of an installation or fabrication related problem with the chosen approach, likely attributed to an uneven fine-tuning of the bias tension in the antagonistic wire setup. To improve reproducibility, automation of production steps is proposed.

In the experiments, the martensite-austenite phase transition was characterised by a transient temperature-driven increase in electrical resistance, followed by a significant drop as a result of the microstructural changes that take place in the process. The austenite-martensite transition shows reversed behaviour. This phenomenon is referred to as the intrinsic sensing capability of SMA, which in principle allows the state of the actuator to be read from its resistance. Based on this, applications with electrical feedback can be realised, e.g., visual and haptic feedback for the surgeon on the current deflection status of the instrument.

The device temperature has a significant influence on the operating behaviour of the developed system. The course of the maximum deflection during warm up showed an initial increase and later a decrease while the wire forces rose until they reached a plateau. This is due to the rising operating temperatures of the device. The wire used requires a temperature below 50 °C to almost fully reach the martensite state in each grain^40^. When the overall device temperature surpasses the martensite finish temperature, incomplete phase transformation occurs, leading to wire shortening and increased elongation of the tension springs. This systematic effect superimposes on TRIP and stress relaxation in the SMA wires, which are negligible in the context of the experimental setup (short lifetime and small number of cycles), but must be taken into account for further developments. SMA wire relaxation can occur in the long term over cycles due to the elongation of the wire arising from transformation induced plasticity leading to less forces from the spring bias.

Considering the parallel progression of the temperature, deflection and force readings, the incomplete phase transformation becomes apparent. A slight increase in device temperature contributes to a more complete phase transition process in the given time, suggesting that longer cycle times would yield even better results. In contrast, an excessively high device temperature prevents the material phases in the deactivated wire from fully resetting, which restricts the deflection of the joint by the active wire. The change in the thermal operating point of the antagonistic wire arrangement also affects the shape of the force curves in Fig. 8c. The activated wire reaches relevant transition temperatures in a shorter time, whereas the deactivated wire returns to the martensite phase more slowly. This asymmetry results in increasing interference between the austenite states of the two wires. This is especially evident in the later force cycles at the peaks and bumps immediately after switching the excitation current on or off. These features move closer to the activation times and become more pronounced. This highlights the importance of selecting and optimising the transition temperatures of the SMA wires appropriately depending on the application. Moreover, these results call for a solution to the thermal problem as a whole.

The heat input at the instrument shaft is irrespective of the position, as both the SMA wire diameter and the electrical current along the shaft are constant. However, the temperature at the tip of the instrument was consistently higher than at the base. This can be explained by the solid aluminium body, which acts as a heat sink on the lower side.

Based on the shape of the curves in Fig. 10, the thermal inertia of the SMA actuator system can be observed. A high actuation frequency results in a more elliptical hysteresis shape with lower maximum deflection values. As anticipated, the good agreement of the cycles of 120 s and 300 s shows that the dynamic variance and inertia of the system parameters diminish with slower actuation frequencies. The hysteresis loop becomes narrower as the activation frequency decreases. This is because the deactivated wire has more time to cool down, reducing the counterforce that the activated wire must overcome. As the overall device temperature remains constant due to the constant RMS value of the input current, the observed mechanical behaviour is temperature-independent. In a cooler state, the temperature-related phenomena that affect the deflection angles are expected to decrease, resulting in larger angles, faster cooling of the deactivated wire and thus a narrower hysteresis loop.

The results in Fig. 9b indicate the impact of the excitation frequency on the maximum achievable deflection. If the inputs are too fast, the antagonist wire cannot cool down passively within the specified time, which means that the maximum deflection decreases as the actuation frequency increases. Without active cooling, both the reaction speed and the wire forces are determined by the wire thickness, whereby the two parameters interfere with each other. The SMA wires used provide a good balance between dynamic response and maximum joint deflection.

In continuous operation with activation currents of 175% of the rated current, all devices exceeded the set temperature limit of 50 °C. In future applications, the permanently extreme activation times, currents and deflections demonstrated are not to be expected, which will have a positive effect on the device temperature. Nevertheless, special attention must be paid to optimising the device temperature in further developments. As the antagonistic design has an optimal thermal operating point, the performance of future prototypes could be increased by integrating an active cooling system. Fluid cooling is a possibility in the existing installation space with additional channels, and this should be the subject of future investigations.

From the measurement results and Ohm’s law, maximum dc voltages of approx. 15.75 V can be calculated, which are considered safe for humans. In addition, the design also enables complete isolation of the electrically connected parts so that there is no risk of voltage being applied to external components. However, the use of an elastic insulating sleeve would further improve the electrical and thermal insulation and protect the device from leaks in a real surgery setting. Furthermore, the influence of the SMA wire length and the activation currents on the temperature and fatigue behaviour should be investigated. The actuation speeds and deflection behaviour achieved are promising for a laparoscopic camera system, but further testing in a real surgical environment is needed to assess the clinical suitability of the frequency and deflection results for surgical instruments with high motion dynamics.

The built-in camera combined with electronic controllability offers the potential to further implement digital assistance functions, such as position sensing and automatic target tracking. This could be of major significance in advancing operation outcome and surgeon performance as well as training time. A more advanced version of the instrument design presented needs to be tested by surgeons in animal experiments.

A first digital assistance feature, the deflection hold function, has already been successfully implemented. This function equates to the mechanical clamping mechanism used in endoscopes with Bowden cables. However, it required no additional mechanical parts and was implemented by simply reprogramming the reusable control unit. This underlines the advantages of electronically controlled instruments compared to purely mechanically driven surgical tools. The developed actuator concept is transferable to other flexible instruments, as the actuator diameter can be easily changed, the electrical control can be adopted and the effects found can be managed. It is plausible to collaborate with medical experts to implement the SMA actuator platform in other novel endoscopic instruments. It is necessary to create a consistent analytic model of the antagonistic, spring-tensioned approach to achieve these objectives.

## Conclusion

This work demonstrates the feasibility of a SMA-based, spring-tensioned, and antagonistic wire system for the electric control of a flexible-tip laparoscope through an open-loop approach under laboratory conditions. SMA wires have the potential to actuate not only laparoscopes but also endoscopes and endoscopic instruments due to their geometry. With roll-to-roll production, for example multi-material coextrusion, they offer a low-cost option for an actuator platform.

The developed system enables the precise deflection of a mounted video camera. The instrument is controlled manually with a thumb joystick or automatically via a digital interface. Combined with the use of suitable sensors or the intrinsic sensor properties of SMA, this offers great potential for the realization of advanced, feedback-based, and cyber-medical assistance functions. The control electronics developed are external to the instrument and can be reused with multiple devices. The actuator design is suitable for scaling and transfer to other applications and the potential realization of disposable medical instruments. Essential electromechanical performance parameters were determined and wear and life phases of the novel SMA setup could be identified, which serve as optimisation approaches for future work. A few limitations need to be overcome in the further development of the proposed system. This includes focus on scaling, mechanical optimisation, and the regulation of the device and wire temperatures. The latter is crucial, as the measured temperatures were the only requirement that exceeded the specifications, and the SMA performance decreases as the maximum device temperature is approached.

## Electronic supplementary material

Below is the link to the electronic supplementary material.

Supplementary Material 1

## Data Availability

The data collected and analysed in this study are available from the corresponding authors upon reasonable request.
